# Colloidal InAs Quantum Dot‐Based Infrared Optoelectronics Enabled by Universal Dual‐Ligand Passivation

**DOI:** 10.1002/advs.202306798

**Published:** 2024-01-19

**Authors:** Min‐Jae Si, Seungin Jee, Minjung Yang, Dongeon Kim, Yongnam Ahn, Seungjin Lee, Changjo Kim, In‐Ho Bae, Se‐Woong Baek

**Affiliations:** ^1^ Department of Chemical and Biological Engineering Korea University Seoul 02841 Republic of Korea; ^2^ Department of Energy Engineering Korea Institute of Energy Technology (KENTECH) Naju 58330 Republic of Korea; ^3^ Nanotechnology and Advanced Spectroscopy Team, C‐PCS, Chemistry Division Los Alamos National Laboratory Los Alamos NM USA; ^4^ Division of Physical Metrology Korea Research Institute of Standards and Science Daejeon 34113 Republic of Korea

**Keywords:** colloidal quantum dots, infrared, photodetector, photomultiplication, surface passivation

## Abstract

Solution‐processed low‐bandgap semiconductors are crucial to next‐generation infrared (IR) detection for various applications, such as autonomous driving, virtual reality, recognitions, and quantum communications. In particular, III–V group colloidal quantum dots (CQDs) are interesting as nontoxic bandgap‐tunable materials and suitable for IR absorbers; however, the device performance is still lower than that of Pb‐based devices. Herein, a universal surface‐passivation method of InAs CQDs enabled by intermediate phase transfer (IPT), a preliminary process that exchanges native ligands with aromatic ligands on the CQD surface is presented. IPT yields highly stable CQD ink. In particular, desirable surface ligands with various reactivities can be obtained by dispersing them in green solvents. Furthermore, CQD near‐infrared (NIR) photodetectors are demonstrated using solution processes. Careful surface ligand control via IPT is revealed that enables the modulation of surface‐mediated photomultiplication, resulting in a notable gain control up to ≈10 with a fast rise/fall response time (≈12/36 ns). Considering the figure of merit (FOM), EQE versus response time (or −3 dB bandwidth), the optimal CQD photodiode yields one of the highest FOMs among all previously reported solution‐processed nontoxic semiconductors comprising organics, perovskites, and CQDs in the NIR wavelength range.

## Graphical abstract

Surface passivation using intermediate phase transfer (IPT) enhances the performance of InAs colloidal quantum dot (CQD) photodetectors. IPT enables stable CQD ink in green solvents and facile control over the surface condition of the nanocrystals. The resultant photodiodes exhibit high gain (≈10) and fast response times (≈12/36 ns), resulting in a notable figure of merit (FOM).

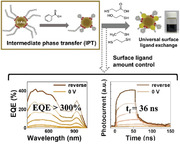

## 1 Introduction

The detection of infrared (IR) light is crucial for various applications such as autonomous driving,^[^ ^1^ ^]^ bio‐imaging,^[^ ^2^ ^]^ and recognition.^[^ ^3^ ^]^ Solution‐processed low‐bandgap semiconductors have received considerable attention as IR absorbers^[^ ^4^ ^]^ owing to the associated low‐cost synthesis methods and scalable processes^[^ ^5^ ^]^; furthermore, they exhibit lightweight characteristics and various form factors.^[^ ^6^ ^]^

A colloidal quantum dot (CQD), a semiconducting nanocrystal, is a promising material owing to its quantum‐confined bandgap tunability across visible to shortwave‐infrared (SWIR) wavelengths.^[^ ^7^ ^]^ However, many of the IR CQDs studied previously consisted of toxic heavy metals, such as Hg, Cd, and Pb, thus limiting their commercial viability.^[^ ^8^ ^]^ Most high‐performance IR optoelectronic devices use PbS and HgTe CQDs.^[^ ^9^ ^]^

InAs (or Sb) CQDs have been recently investigated because of the compliance of restriction of hazardous substances (RoHS) and the low‐bulk bandgap (0.17–0.35 eV) properties of these CQDs.^[^ ^10^ ^]^ Specifically, the low permittivity of an InAs CQD enables the fabrication of ultrafast optoelectronics.^[^ ^11^ ^]^ However, the InAs CQD has several limitations. First, the quantum efficiency of InAs photodetectors reaches only ≈30%,^[^ ^12^ ^]^ which is significantly lower than that of Pb‐based devices (≈80%)^[^ ^9b^ ^]^ owing to a lack of surface passivation studies and unoptimized fabrication processes.^[^ ^13^ ^]^ Second, toxic solvents such as *N*,*N*‐dimethylformamide (DMF) are typically used to prepare the CQD ink, which can be a potential limiting factor in manufacturing processes.^[^ ^14^ ^]^

Herein, we present an intermediate phase transfer (IPT) strategy to overcome the aforementioned challenges. The IPT method uses aromatic organic ligands on the InAs CQD surface to first exchange native oleic acid (OA) and transfer the phase to less polar solvents for CQD ink stabilization.^[^ ^15^ ^]^ Desirable short ligand compounds can then be attached carefully to the CQD surface to adjust the electronic structure and form conductive CQD solids. IPT enables the utilization of various organic ligands exhibiting low to high reactivity on the CQD surface and dilution of the CQDs in various green solvents, such as 2‐methyltetrahydrofuran (2‐meTHF)^[^ ^16^ ^]^ and 2‐methylanisole (2‐MA).^[^ ^17^ ^]^ The resultant CQD ink exhibits high‐colloidal stability, which is 25‐fold higher than that of CQD inks using conventional ligand exchange.

Furthermore, we fabricated an InAs CQD‐based diode‐type near‐infrared (NIR) photodetector. Careful control of the CQD surface via IPT enables the modulation of surface‐mediated photomultiplication, thus boosting the gain and responsivity. Especially, surface‐mediated photomultiplication minimizes the trade‐off between gain and response time, which results in both an EQE of 292% and rise/fall time of 12.4/36.1 ns at the reverse bias. We validate our results using an important performance metric for the photodetector: quantum efficiency versus response time^[^ ^18^ ^]^ as a figure of merit (FOM) and highlight that, to the best of our knowledge, our results constitute one of the best performances in NIR photodetectors among all previously reported solution‐processed nontoxic materials, comprising organics, Pb‐free perovskites, and Pb‐free CQDs.

## 2 Results and Discussion

### 2.1 Stable InAs CQD Green Ink Using IPT

Several surface modification strategies for InAs CQDs have been proposed previously to fabricate conductive CQD solids.^[^ ^19^ ^]^ To this end, highly reactive ligands or surface etchants were used to break inherent covalent bonding and substitute the native oleate ligands. These methods are facile for exchanging the surface ligands of InAs CQDs. However, careful surface control using low‐reactive ligands and co‐passivation using multiple ligands for passivating fractional dangling bonds efficiently is challenging^[^ ^20^ ^]^

**Figure 1:**
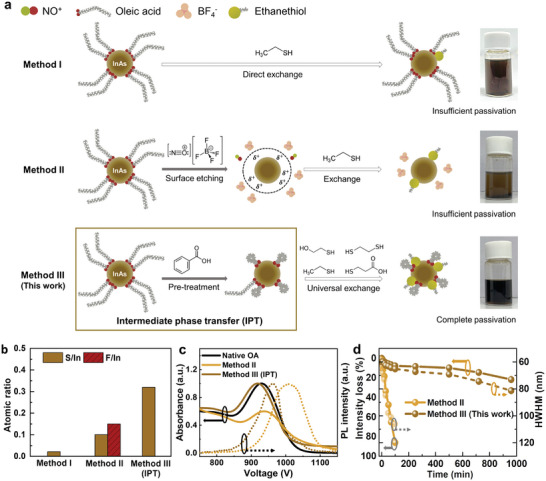
Surface modification of InAs colloidal quantum dots (CQDs) using the intermediate phase transfer (IPT) method. a) Schematics of three ligand exchange methods using InAs CQD and the corresponding photographic images of the resultant CQD ink: i) direct ligand exchange method, ii) surface etching method using NOBF_4_, and iii) IPT. All photographs exhibit the ethanethiol (ET)‐capped CQD ink dissolved in 2‐methylanisole (2‐MA). b) X‐ray photoelectron spectroscopy (XPS) atomic ratios (ratios of sulfur to indium (brown) and fluorine to indium (red)) of thiol‐exchanged CQD surfaces using various ligand exchange methods. c) Normalized absorbance (solid line) and PL (dashed line) spectra of CQD inks fabricated by methods II (yellow‐brown) and method III (brown), compared to the absorbance spectrum of native OA ligands (black, solid). d) Intensity loss (line) and HWHM (dashed) were measured using the absorbance spectra as a function of time for the CQD inks obtained using methods II (yellow‐brown) and III (brown).

**Figure** **1**a depicts two previously reported ligand exchange methods for InAs CQDs. We performed X‐ray photoelectron spectroscopy (XPS) to verify the surface passivation of the CQDs (Figure 1b). In this study, thiol‐ligands with various reactivities, such as 2‐mercaptoethanol (2‐ME), 1,2‐ethanedithiol (EDT), and ethanethiol (ET) were prepared in the DMF phase.^[^ ^12^ ^]^ For the direct ligand exchange process (method I), the thiol‐ligand precursor solution was mixed with the oleic acid (OA)‐capped InAs CQDs (Figure S1, Supporting Information), resulting in the solution phase transfer from the nonpolar to polar DMF phase. A direct ligand exchange from OA to thiols is simple and facile; however, low‐reactivity ligands, such as ET, cannot attach effectively to the CQD surface (Figure S2, Supporting Information). Therefore, the colloidal stability of the CQDs dissolved in polar solvents is reduced considerably over time (Figure 1a, top).

Subsequently, we performed surface etching (method II). The surface etchant, nitrosyl tetrafluoroborate (NOBF_4_), detaches OA ligands and removes oxidized layers from the InAs CQD surface.^[^ ^21^ ^]^ We performed ET ligand passivation after NOBF_4_ etching and observed that it shows a higher ratio of sulfur atoms bound to the CQD surface than method I, indicating that the surface etching facilitates thiol ligand exchange (Figure 1b). However, a notable amount of fluorine is also observed, presumably indicating the presence of BF_4_^−^ residues on the CQD surface even after several times of rinsing. Thus, aggregations and precipitations were observed after redispersion in weakly polar green solvents, such as 2‐MA (Figure 1a, middle).

In this study, we devised a universal ligand exchange method using IPT (method III). Molecules with short‐chain carboxylates can facilitate the ligand exchange process of CQDs by controlling steric hindrance.^[^ ^22^, ^23^ ^]^ Therefore, IPT was performed using benzoic acid (BA) ligands to exchange the native OA ligands and transfer a solution phase from nonpolar to weakly polar solvents; subsequently, various thiol ligands were adjusted. We selected BA ligands for IPT because polarizing the aromatic ring produces a stable CQD ink in weakly polar solvents and enhances the CQD–solvent interactions.^[^ ^15^ ^]^ Furthermore, the dispersion of CQDs in less‐polar solvents maintains stable ink stability during secondary thiol ligand passivation. To this end, we utilized various polar solvents such as 2‐methyltetrahydrofuran(2‐meTHF), 2‐methylanisole (2‐MA), and chlorobenzene with dielectric constants ranging from 2.3 to 7.0, which is sufficient to shield charges and prevent CQD aggregation.^[^ ^24^ ^]^ The reactivity of the thiol ligands was considered when choosing the ligand precursors, to prevent excess substitution of BA ligands that stabilize the CQD–solvent interaction, forming stable CQD ink. As a result, IPT enables the effective attachment of various thiols on the CQD surface, justified by the highest sulfur‐to‐indium (S/In) ratio (Figure 1b). Previously, various co‐passivation strategies were utilized for III‐V CQDs by treating anions (halides) and amines.^[^ ^25^ ^]^ Most of the prior studies focus on X‐type and Z‐type co‐passivation which usually employ halide‐amine or amphoteric halide ligands.^[^ ^26^ ^]^ The IPT strategy utilized dual X‐type, aromatic, and thiol, ligands to co‐passivate the CQD surface for achieving both efficient surface passivation and high colloidal stability. Furthermore, it exhibits a stable CQD ink in various green solvents (i.e., 2‐meTHF and 2‐MA), derived from renewable resources^[^ ^16^, ^27^ ^]^ (Figure S3, Supporting Information).

We evaluated absorbance and photoluminescence (PL) measurements to investigate the stability of ET‐passivated InAs CQD ink for various methods (Figure 1c). Method I was not characterized because surface passivation was not demonstrated adequately therein (Figure 1b; Figure S2, Supporting Information). Since NOBF_4_ (method II) and BA (method III) ligands are acids, the removal of In‐oleate ligands via surface etching is expected for both methods.^[^ ^28^ ^]^ The IPT‐treated CQD presents a blue shift of absorbance peak, indicating the size decreases due to the surface etching. Given that NOBF_4_ has been reported to be a stronger acid (*pKa*: −0.4–3.4)^[^ ^29^ ^]^ compared to BA (*pKa*: 4.19),^[^ ^30^ ^]^ it should exhibit increased blue‐shift of the absorption peaks. However, the CQD ink treated with method II shows red‐shifted and broader absorbance and PL spectra than those of IPT (method III) The stronger acid‐induced surface damage^[^ ^31^ ^]^ and insufficient attachment of thiol ligands due to BF_4_^−^ residues resulted in CQD aggregation, causing the poor colloidal stability (Figure 1a).

In addition, method II yields a higher Stokes shift (97 meV) than that of method III (49 meV), indicating the lower colloidal stability caused by CQD aggregation and defect‐induced mid‐gap states^[^ ^32^ ^]^ after surface etching with NOBF_4_.

We also examined the absorption intensity loss and a half‐width‐at‐half‐maximum (HWHM) of the InAs CQD ink over time (Figure 1d). In the case of method II, the absorbance intensity decreases to half of the initial value after only 30 min because of CQD aggregation. In contrast, the use of the CQD ink in conjunction with IPT retains >90% of its initial intensity and yields a low HWHM (<70 nm) for periods of up to 500 min (Figure S4, Supporting Information). Furthermore, the PL quantum yield increased from 1.11% to 1.32% after ligand exchange via IPT, proving that efficient surface passivation decreased surface trap states.^[^ ^33^ ^]^ In summary, IPT is a promising strategy for achieving improved passivation with various ligands, which results in high colloidal stability in various solvents.

### 2.2 Surface Characterizations of IPT‐Treated InAs CQDs

**Figure 2:**
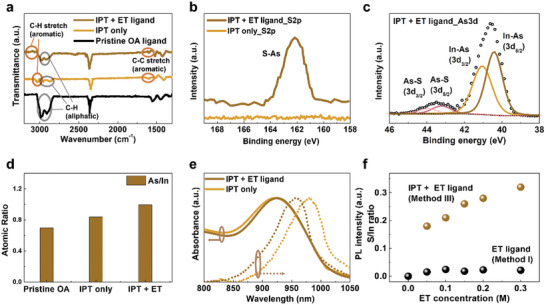
Surface characterization of InAs CQDs with IPT. a) Fourier‐transform infrared spectroscopy (FTIR) measurements of InAs CQD solids using pristine oleic acid (OA) ligands (black), only IPT (dark orange), and ethanethiol (ET) ligands in conjunction with IPT (brown). b) XPS S 2p spectra of CQDs using ET ligand with IPT (brown) and only IPT (dark orange). c) XPS As 3d spectra of CQDs with ET ligands. d) As/In atomic ratio of pristine OA‐capped CQD (left), and IPT‐treated CQD inks before (middle) and after adding ET ligands (right). e) Normalized absorbance and PL spectra of CQD ink with only IPT (dark orange) and inks treated with ET ligands after IPT (brown). f) S/In ratio of ET‐treated CQD solids with (brown) and without IPT (black) depending on the concentration of the ET ligand precursor solution.

We studied the surface properties of IPT‐treated InAs CQDs using Fourier‐transform infrared spectroscopy (FTIR) (**Figure** **2**a). OA‐passivated InAs CQDs clearly show aliphatic C‐H bonding peaks at 2910 and 2980 cm^−1^. After the application of IPT, peaks corresponding to aliphatic C‐H bonding are reduced considerably, and peaks at 3050 and 1600 cm^−1^ appear, which correspond to the vibrations of C‐H and C‐C stretching in the aromatic rings, respectively, indicating that OA ligands are partially exchanged by BA ligands^[^ ^15^ ^]^ (Figure S5, Supporting Information). The peaks of aliphatic C‐H bonds appear again after the addition of ET ligands, justifying the ET ligand passivation on the surface of the InAs CQDs.^[^ ^12^ ^]^ At the same time, the peaks related to aromatic C‐H stretch slightly reduced, which indicates partial substitution of BA ligands to ET ligands. A few residual peaks near 3000 cm^−1^ corresponding to C‐H stretching vibration remain because of the carboxylic groups in the BA ligands.

We conducted XPS to analyze further the elemental compositions of the InAs CQDs (Figure 2b,c). A peak signal at 162 eV appears in the S 2p spectra after ET ligand exchange when the IPT method is used; this finding implies that the thiolate was bounded on the surface of the InAs CQD in Figure 1b.^[^ ^34^ ^]^ We also compared the As 3d spectra of the InAs CQDs before (Figure S6, Supporting Information) and after (Figure 2c) the ET ligand exchange using IPT and confirmed that a peak corresponding to the As‐S bond appeared at 43.5 eV after the ET ligand passivation; this finding implies that the ET ligands form bonds with the As atoms, which is consistent with previous reports^[^ ^35^ ^]^ (Figure 2c).

We also compared the As/In atomic ratio of IPT‐treated InAs CQD (Figure 2d). The As/In ratio increases from 0.697 to 0.837 after IPT and further to 0.873 as ET ligands are subsequently added because In‐oleate ligands are detached by the incoming ligands.^[^ ^12^ ^]^ This indicates that thiol ligand exchange further reconstructs the CQD surface, resulting in a more balanced stoichiometry.^[^ ^11^ ^]^ Regarding the S/In ratio after ET ligand exchange process in Figure 1b, sulfur only exists for thiol‐exchanged CQD after IPT, as revealed in previous discussions. The surface analysis indicates that BA ligands are attached to the InAs CQD surface during the IPT followed by the attachment of ET ligands, resulting in the co‐passivation on the CQD surface.

The absorbance and PL were characterized to ensure the co‐passivation quality using IPT (Figure 2e). Both the absorbance and PL exhibit clear optical features at 918 and 961 nm, respectively. Following the addition of ET ligands, both spectra become narrower, and the Stokes shift decreases from 75 to 49 meV owing to the passivation of the CQD surface. A small blue shift in absorbance is observed after the ET ligand exchange presumably because of the decrease in the effective size after the removal of the In‐oleate ligands (Figure 2d).^[^ ^12^ ^]^

Finally, we examined whether the extent of surface passivation can be modulated with the concentration of the ET ligands. The XPS results show that the degree of surface passivation does not change significantly with the ET ligand concentration for method I; by contrast, the extent of passivation can be adjusted using IPT (Figure 2f). Beyond the efficient surface passivation strategy, IPT may be used to control the electrical properties of InAs CQD solids. This implies that the physical properties of optoelectronics can be effectively modulated.^[^ ^36^ ^]^

### 2.3 InAs CQD NIR Photodetector

**Figure 3:**
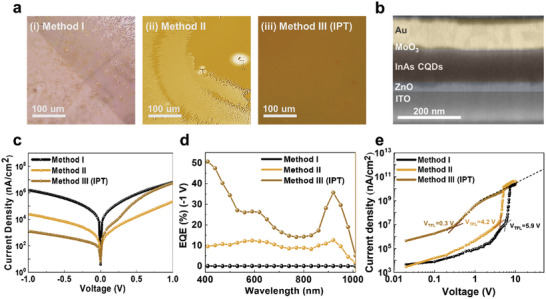
NIR photodetector performance depends on the ligand exchange method of InAs CQD. a. optical microscope image of CQD solid thin film treated with the method i) I, ii) II, and iii) III (IPT). b. Cross‐sectional image of InAs CQD photodiode device scanned by SEM. c. J‐V characteristics of InAs CQD photodiodes based on method I (black), II (yellow‐brown), and III (IPT) (brown). d. EQE spectra of CQD device at ‐1 V with method I (black), method II (yellow‐brown), and method III (IPT) (brown). e. SCLC characterization of hole‐only devices of the method I (black), II (yellow‐brown), and III (IPT) (brown). An ET ligand concentration of 0.3 M was employed for IPT process.

We fabricated thin InAs CQD solids prepared by IPT and conventional methods (methods I and II) (**Figure** **3**a). Note that an identical concentration of ET ligands (0.3 m) was utilized for all conditions. The CQD film images scanned by optical microscopy show different results at each method the films using methods I and II exhibit cracks and large particles, which could be attributed to the aggregated CQDs. In comparison, employing the IPT method (method III) results in uniform CQD solid thin films after the spin coating. This result indicates that the IPT method allows for better substitution of native OA ligands to thiol and BA ligands compared to conventional methods I and II, leading to enhanced ink dispersion.

We fabricated diode‐type photodetectors using the InAs CQD solids prepared using each ligand exchange method. The overall device structure consists of indium–tin oxide (ITO) as a transparent electrode (≈120 nm), a ZnO electron transport layer (≈30 nm), the InAs CQD IR absorbing layer (≈120 nm), a MoO_3_ (≈10 nm) hole transport layer, and an Au top electrode (≈100 nm) (Figure 3b).

We measured the current density under dark conditions to confirm the rectifying feature of the CQD photodiode. The IPT‐treated InAs CQD device effectively suppresses leakage current and yields a dark current density of 6.8 nA cm^−2^ at 0 V and a rectification ratio >2000 (Figure 3c). However, the CQD photodiodes made with methods I and II demonstrate one to three orders of worse dark current at −1 V, respectively. We attribute the substantial dark current to tunneling leakage current due to insufficient passivation of CQD surface by the method I and II.^[^ ^37^ ^]^ Furthermore, cracks and pinholes in CQD films result in unstable *I*‐*V* characteristics under the higher reverse voltage, reducing operation stability and performance. (Figure S7, Supporting Information). To compare our results, external quantum efficiency (EQE) was examined at each fabrication condition. (Figure 3d) IPT‐treated InAs CQD devices exhibit an EQE of 16% under 0 V and 36% with a bias at −1 V, which is over a three‐fold increase in EQE compared to devices based on method II, and the calculated responsivity is ≈0.27 A W^−1^. (Figure S8, Supporting Information). We attribute the increase in EQE to a higher extraction efficiency attained by the co‐passivation of BA and ET ligands and the resultant uniform film via the use of IPT, as discussed in Figure 2 and Figure 3.

We measured the space‐charge‐limited current (SCLC) of CQD solids to verify the electrical properties at each film fabrication condition^[^ ^38^ ^]^ (Figure 3e). The trap density was obtained to be 3.8 × 10^18^ cm^−3^ for method I, and 1.3 × 10^18^  cm^−3^ for method II, respectively. CQD solids using the IPT method exhibit 7.0 × 10^16^ cm^−3^, indicating two orders of magnitude lower trap density. The IPT method enables an efficient ligand exchange on the CQD surface (Figure 2f) and also demonstrates uniform film morphology (Figure 3a). We, therefore, conclude that both uniform film formation and the improved surface passivation of CQD contribute to suppressing trap density and pinholes, leading to an increase in device performance.^[^ ^23^ ^]^

**Figure 4:**
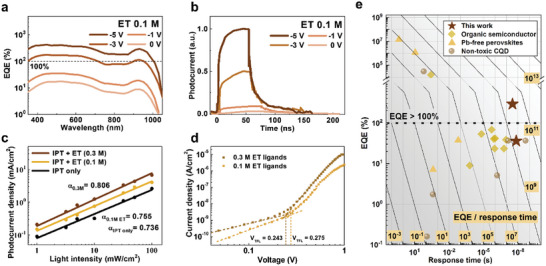
Characterization of surface‐mediated photomultiplication. a. EQE of IPT‐treated InAs CQD photodiodes (ET ligands concentration: 0.1 m) as a function of reverse bias scanned from 0 V (yellow‐brown) to ‐5 V (brown). b. Transient photoresponse of IPT‐treated InAs CQD photodiodes (ET ligands concentration: 0.1 m) as a function of reverse bias from 0 V (light‐brown) to ‐5 V (brown). c. Light‐intensity dependent photocurrent density for IPT‐InAs photodiode: only IPT process (black), 0.1 m of ET ligands (yellow), and 0.3 M of ET ligands (dark brown). d. SCLC characteristics of photodiode device using IPT process with different concentrations of ET ligands: 0.1 m (yellow), and 0.3 m (brown). e. EQE versus response (fall) time of the solution‐processed NIR photodetectors including non‐toxic CQDs (grey circle), Pb‐free perovskites (orange triangle), organic semiconductors (yellow square), and this work (brown pentacle). The grid line indicates EQE/response time used as a figure of merits (FOM).

We also measured the noise current at various operating frequencies without external bias to analyze the factors affecting the noise of the InAs CQD photodiodes: 1/f noise accounts for more than 95% of the total noise current at an operating frequency of 1 kHz (Figure S9, Supporting Information). We obtained the specific detectivities and photocurrent response times of devices (Figure 4b; Figure S10, Supporting Information). The specific detectivity reaches 1.9 × 10^11^ Jones at 930 nm at an operating frequency of 1 kHz, which is comparable to prior CQD NIR photodiodes.^[^ ^25^, ^39^ ^]^ IPT‐based devices exhibit 1 to 2 orders of magnitude higher detectivity at 930 nm compared to methods I and II, respectively, which is attributed to enhanced charge extraction, resulting in increased responsivity. The response time obtained by the fall time is 14 ns, which is comparable with the best outcomes in prior reports for similar device areas (≈0.03 mm^2^)^[^ ^11^ ^]^ (Figure S11, Supporting Information).

### 2.4 Surface‐Mediated Photomultiplication in CQD Solids

To enhance the efficiency, we studied the photodetector performance for various ligand concentrations, because IPT can be used to control surface ligands, as discussed previously in Figure 2f. We fabricated a device using the same structure in Figure 3 and found that the CQD device at a lower ET concentration (0.1 m) yields different EQE responses (**Figure** **4**a). As the reverse voltage increases to −5 V, the EQE increases considerably to values up to ≈290%, thus yielding considerable increases in gain (*G*, >10) corresponding to the high responsivity of 2.19 A W^−1^ (Figure 4a; Table S1, Supporting Information). The photodiode keeps rectifying behavior and photocurrent increases as the reverse voltage is applied. Simultaneously, fast responses yield rise/fall times equal to 12.4/36.3 ns (Figure 4b), resulting in >three‐fold EQE/response time performance metrics than those of the device with the ET (0.3 m concentration). Reduced response time is presumably due to decreased drift time and Resistor‐Capacitor (RC) time, resulting from applied reverse bias.^[^ ^40^ ^]^ On the other hand, devices treated with 0.3 m precursors show negligible changes in response time under the reverse bias due to the presence of parasitic capacitance.^[^ ^41^ ^]^ We concluded that the carrier multiplication was generated based on CQD surface control, which implies surface‐mediated photomultiplication.

Photomultiplication is a rational strategy to gain multiple carriers from an absorbed photon. It is usually observed in photoconductors or phototransistors where the gain is increased as electrons (or holes) are extracted slower than the other types of carriers.^[^ ^42^ ^]^ Among the various photomultiplication methods, trap‐assisted multiplication, which modulates trap states to control carrier extraction, has been studied to realize photomultiplication in photodiode structures^[^ ^43^ ^]^.

We observed higher bimolecular recombination as ET ligand concentration decreased by examining the photocurrent at various light intensities^[^ ^44^ ^]^ (Figure 4c). Since the macroscopic CQD film images with different thiol concentrations show negligible differences, we concluded that the origin of the changed trap densities is the different amounts of ligands attached to the CQD surface. (Figure S12, Supporting Information) We therefore suggest that surface‐mediated multiplication using the IPT method has an analogous mechanism with typical trap‐assisted photomultiplication.^[^ ^45^ ^]^ Compared to conventional methods I and II, the improved quality of CQD thin film by the IPT method enabled high operation stability under reverse bias, which is required for efficient charge injection in trap‐assisted photomultiplication (Figure S7, Supporting Information)^[^ ^46^ ^]^.

The gain and response time of a trap‐assisted photomultiplication‐type photodiode can be obtained using the following equations:^[^ ^47^ ^]^

(1) $$G\;=\frac{\tau_{lifetime}}{\tau_{transit}}\;\left(1+\frac{\Delta n}{\Delta p}\frac{\mu_{n}}{\mu_{p}}\right),\tau_{response}=\frac{p_{t}}{p}\;\tau_{lifetime}$$

where *G* is photoconductive gain, τ_*lifetime*_ is minority carrier lifetime, τ_*transit*_ is carrier transit time, Δ*n* and Δ*p* are excess electron and hole density, µ_*n*_ and µ_*p*_ is the mobility of electrons and holes, τ_*response*_ is the response time of the photodetector, *p_t_* and p is the trapped carrier and free carrier density. Both factors are a function of carrier lifetime; therefore, gain and response time typically have a trade‐off relationship, which implies that an increase in gain results in a slower response time. Because of this compromise, we further considered an important figure of merit, photoconductive gain/response time (or × bandwidth) using τ_*transit*_ = *L* ^2^ /(µ*V*) and Equation (1):^[^ ^48^ ^]^

(2) $$\frac{G}{\tau_{response}}=\frac{p}{p_{t}}\;\left(1+\frac{\Delta n}{\Delta p}\frac{\mu_{n}}{\mu_{p}}\right)\left(\frac{\mu V_{A}}{L^{2}}\right)$$

where *V*_A_ is the externally applied bias, *L* is the length between the electrodes, and µ is the mobility of a faster carrier. Previously, trap‐assisted photomultiplication has been demonstrated by controlling the trap states using interfacial layers.^[^ ^49^ ^]^ Most of the works exhibited notable gain (50–120) but low gain/response time values because of the substantial delayed response time (0.01–5 ms). From Equation (2), we infer that this originates from a high level of trap density (p_t_) at the interfacial layer.^[^ ^50^ ^]^

We studied the trap density of CQD devices using space‐charge‐limited current (SCLC) measurements. (Figure 4d; Table S2, Supporting Information). As a result, we obtained modest increases in trap density (≈12%) by adjusting the ET concentration using IPT method. This results in a lower gain (≈10) compared to previous works (50–120) but a small expense in response time from 14 to 36 ns (vs 0.01–5 ms of previous works). As a result, the overall gain/response time value is increased. We concluded that precise control of the surface trap of CQD using IPT method can be an effective strategy for increasing gain/response in photodiode structure.^[^ ^47b^ ^]^ In particular, we found that the CQD thin films scanned by optical microscope showed similar quality at various ET ligand concentrations, indicating the importance of controlling trap states for efficient photomultiplication.

We obtained the performance metrics of solution‐processed non‐toxic NIR photodetectors. We evaluated previous reports on various types of NIR photodetectors in the range of 850–950 nm, which is an important wavelength region for ranging and biosensing applications.^[^ ^1^ ^]^ In addition, we only considered the reports that presented responsivity (or gain) as well as temporal response time (or bandwidth). We plotted the EQE versus response time as the FOM (Figure 4e). Note that EQE versus 3 dB bandwidth results are also displayed in Figure S13, Supporting Information. The IPT‐InAs CQD‐based photodiode exhibits both high‐quantum efficiency and fast response time, which exhibits one of the highest FOMs among all the solution‐processed non‐toxic NIR photodetectors comprising CQDs, organics, and perovskites (Table S3, Supporting Information).

## 3 Conclusions

In this study, we demonstrated a new ligand exchange strategy that facilitated thiol ligand exchange by adjusting the co‐passivation of InAs CQDs. IPT improved colloidal ink stability and surface passivation. Moreover, it enabled a fine control of the amount of thiol ligand bound to the CQD surface and resulted in surface‐mediated photomultiplication. This enabled the control of the gain of the photodiode with a low response time, thus yielding promising EQE versus response time (or bandwidth) performance metrics. Surface‐mediated multiplication via IPT offers a strong advantage to CQD‐based devices because CQD solids have significantly higher surface‐to‐volume ratios than typical bulk semiconductors. We expect that higher external reverse voltage, more precise control of the trap density, and improved control of the excess carrier kinetics will enable higher FOM for photodiodes. In summary, those advances in the photodetector performances highlight the potential of the universal ligand exchange method for fabricating efficient IR CQD optoelectronics using RoHS‐compatible materials.

## 4 Experimental Section

### Materials

Indium acetate (In(OAc), 99.99%, Sigma‐Aldrich), oleic acid (OA, 90%), hexane (95% anhydrous, Sigma‐Aldrich), 2‐methyltetrahydrofuran (2‐meTHF, 99.0% anhydrous, Sigma‐Aldrich), 2‐methylanisole (2‐MA, 99.0%, Sigma‐Aldrich), toluene (99.5%, Sigma‐Aldrich), 1,2‐ethanedithiol (EDT, 99.0%, Sigma‐Aldrich), ethanethiol (ET, 97%, Sigma‐Aldrich), chlorobenzene (CB, 99.8% anhydrous, Sigma‐Aldrich), acetonitrile (ACN, 99.5%, Sigma‐Aldrich), ethyl acetate (EA, 99.5%, Sigma‐Aldrich), chloroform (CF, 99.8%, Sigma‐Aldrich), butylamine (BTA, 99.5%, Sigma‐Aldrich), benzoic acid (BA, 99.5%, Sigma‐Aldrich), nitrosyl tetrafluoroborate (NOBF_4_, 95%, Sigma‐Aldrich), and dioctylamine (DOA, 97%, Sigma‐Aldrich) were purchased and used as received. The compounds 1‐octadecene (ODE, 90%, Alfa Aesar), octane (98%, Alfa Aesar), and 3‐mercaptopropionic acid (MPA, 99%, Alfa Aesar) were purchased and used without further purification. 1‐butanol (BuOH, 99.8%) was purchased from Junsei chemical, ME (99%) was purchased from Daejung, and Tris(trimethylsilyl)arsine ((TMSi)_3_As, 99%) was purchased from JSI Silicone and was distilled before use.

### Synthesis of InAs CQD

InAs CQDs were synthesized using a modified method based on a previous study.^[^ ^21^ ^]^ The process was conducted by continuously injecting amorphous InAs clusters into InAs seeds. For the synthesis of the InAs seeds, a mixture of In(OAc)_3_ (0.29 g, 1 mmol) with oleic acid (0.85 g, 3 mmol) in ODE (5 mL) was added to a 100 mL three‐neck round‐bottom flask degassed at 120 °C for 2 h under vacuum conditions. (TMSi)_3_As (0.14 g, 0.5 mmol) mixed with DOA (0.36 g, 1.5 mmol) in 1 mL degassed ODE was maintained at 60 °C for 15 min in a glovebox to fabricate the As solution for the InAs seeds. The As solution was retained until the solution turned brown and was injected into the In(OAc)_3_ solution at 300°C. The seeds of InAs were grown in the mixed solution at 300 °C for 20 min. For the synthesis of the InAs clusters, In(OAc)_3_ (1.74 g, 6 mmol) mixed with OA (5.10 g, 18 mmol) in ODE (30 mL) was added to a 100 mL three‐neck round‐bottom flask degassed at 120 °C for 2 h in a vacuum. (TMSi)_3_As (0.84 g, 3 mmol) mixed with DOA (2.17 g, 9 mmol) in 6 mL degassed ODE was maintained at 60 °C for 15 min in a glovebox to produce the As solution for clusters. The solution was then injected into the In(OAc)_3_ solution at room temperature and then stirred for 15 min. The InAs cluster solution was loaded in a syringe (diameter = 15.9 mm) and continuously injected at a rate of 2 mL/h into the InAs seed solution at a temperature of 300°C. The injection of the cluster solution was continued for 4–5 h to fabricate a batch of InAs CQDs, which exhibited the first exciton peak at a wavelength of 930 nm. The synthesized CQD solution was precipitated after the addition of butanol and centrifugation at 6000 rpm for 5 min. The precipitate was dispersed in 10 mL of hexane and reprecipitated two more times. Finally, the precipitate was dried in a vacuum overnight and redispersed in octane at a concentration of 20 mg mL^−1^.

### Device Fabrication

ZnO sol‐gel was deposited on washed ITO/glass substrates. The CQD inks in 2‐meTHF (200 mg mL^−1^) were spin‐coated (1500 revolutions per minute for 30 s) on the substrate in the N2‐filled glove box. MoO3 as a hole transport layer (10 nm) and Au (120 nm) were deposited via thermal evaporation. In IPT, the InAs CQDs at 930 nm were used to fabricate the CQD inks. The ligand exchange process was conducted in a N2‐filled glove box. OA‐CQDs were prepared via dissolution in 2‐meTHF (20 mg mL^−1^). For surface reconstruction using the intermediate phase, a ligand solution was prepared by dissolving benzoic acid (BA) ligands in 2‐meTHF. A volume of 1 mL of the BA ligand solution was added dropwise to 1 mL of the InAs CQD solution (20 mg mL^−1^ in 2‐meTHF) with gentle stirring. The mixed solution was vortexed for 10 min, and 6 mL of hexane was added to precipitate the exchanged CQDs followed by centrifugation at 3000 rpm for 2 min. The precipitated CQDs were redispersed in 1 mL of 2‐meTHF. Thiol ligand solution was prepared by dissolving ethanethiol in 2‐meTHF (0.1–0.3 m). The thiol solution was added slowly to the redispersed CQDs and vortexed for 3 min. For the purification, the CQD inks were precipitated by adding hexane and redispersed in 2‐meTHF. The purification steps were repeated twice. The final product was then dried in a vacuum for 30 min and dispersed in the desired solvents.

### Absorbance and PL

For the absorbance analysis, a UV–Visible NIR spectrophotometer (Agilent Technologies, Cary 5000) was used. Steady‐state PL measurements were conducted using a monochromatized Xe lamp as the excitation source, and emission photons were measured using a spectrometer (Hitachi, F‐7000). PLQY was tested by using the Edinburgh FS5 spectrofluorometer with an integrating sphere.

### XPS analysis

XPS spectra were acquired in N2 conditions using a Thermofisher Scientific Nexsa with an Al Kα source. The CQD films were prepared onto Si substrates. Scans were conducted at 0.1 eV with a 50 eV pass energy. The atomic ratios were obtained by integrating the area of each peak and scaled by atomic sensitivity factors. The area of all elements was normalized using the area of In to obtain accurate atomic ratios.

### FTIR Spectroscopy

FTIR measurements were performed using a Perkin–Elmer Spectrum GX, 400–4000 cm‐1 with ATR (Attenuated Total Reflection) accessories. ATR mode measures a sample pressed against a prism with infrared light reflected in the prism.

### Field Emission‐SEM (FE‐SEM) Measurements

Cross‐sectional FE‐SEM was conducted using Helios H5 UC FEI to measure the thickness of the device.

### J–V and Space‐Charge‐Limited Current (SCLC)

Current‐voltage characteristics were measured with a Keithley 2401 source meter unit under dark and illuminated conditions under ambient conditions. The J–V curves were swept from −1.0 V to + 1.0 V at 0.02 V internal steps with 50 ms delay times at each step. The curves were measured with 0.1 V internal steps when swept from −5.0 V to + 5.0 V. For illumination condition, 1 sun (100 mW cm^−2^) was irradiated to characterize the optoelectronic property.

For the SCLC measurements of the hole‐only device, the device was fabricated using ITO/MoO_3_/InAs CQD/MoO_3_/Ag. The current was obtained from the dark J‐V measurements from 0.05 to 10 V. For the SCLC measurements of the whole device, the current was obtained from the dark J–V measurements swept from 0.05 to 1.0 V. The SCLC was fitted to obtain the trap density (N) and trap‐filled limited voltage (V_TFL_) of the devices using the following equation:

(3) $$N_{t}=\frac{2\varepsilon \varepsilon_{0}}{eL^{2}}\;V_{TFL}$$

where ε is the dielectric constant, ε_0_ is the vacuum permittivity, e is the amount of Coulombic charge of the electrons, and L is the thickness of the active layer which was 80–120 nm.

### External Quantum Efficiency (EQE)

EQE spectra were acquired using the QuantX‐300 measurement system (Newport). Monochromatic white light from a Xenon lamp (400 W) chopped by 220 Hz frequency was illuminated at the device, and spectral responses were calculated from the measured photocurrent. The EQE spectrum was used to calculate the responsivity (R) of a photodetector.

(4) $$R\;=\;\frac{EQE\cdot \lambda }{1240}$$

### Noise Equivalent Power (NEP) and Specific Detectivity

The average noise current was measured by connecting the photodiode to an SR570 trans‐impedance preamplifier and an SR830 lock‐in amplifier for converting noise voltage to current. Noise measurements were conducted at 298 K without illumination at various frequencies. Noise current (I_N_) was calculated from the measured noise voltage readout (V_N_) and sensitivity of the preamplifier (S) using the following equation:

(5) $$I_{N}\;\left(A/Hz^{1/2}\right)\;=\;V_{N}\;\left(V/Hz^{1/2}\right)\;\times \;S\;\left(A/V\right)$$

The NEP was calculated by measuring noise current and responsivity.

(6) $$\mathrm{NEP}\;=\frac{I_{N}}{R}\;$$

Specific detectivity (D*) was then calculated using NEP, responsivity, and device area (A, 0.8 mm^2^), based on the following formula:

(7) $$D^{*}=\frac{R\sqrt{A}}{I_{N}}\;$$

The temporal response of the photodetectors was evaluated by measuring the transient photocurrent recorded with a 500 MHz oscilloscope (DSO7054A, Tektronix, input impendence 50 Ω), and a 940 nm 10 ns pulsed laser (MDL‐NS‐940, CNI laser) with 10 kHz repetition rate was used to generate the illumination light.

## Conflict of Interest

The authors declare no conflict of interest.

## Supporting information

Supporting Information

## Data Availability

The data that support the findings of this study are available from the corresponding author upon reasonable request.
