# Supplementary material for: Colloidal InAs Quantum Dot‐Based Infrared Optoelectronics Enabled by Universal Dual‐Ligand Passivation
Source: Adv Sci (Weinh). 2024 Jan 19;11(13):2306798. doi: 10.1002/advs.202306798 (PMC10987160; doi:10.1002/advs.202306798)
Supplement: Supplementary file 1 — Supporting Information [file ADVS-11-2306798-s001.pdf]

ADVANCED SCIENCE
Open Access

Supporting Information

for *Adv. Sci.*, DOI 10.1002/advs.202306798

Colloidal InAs Quantum Dot-Based Infrared Optoelectronics Enabled by Universal Dual-Ligand Passivation

Min-Jae Si, Seungin Jee, Minjung Yang, Dongeon Kim, Yongnam Ahn, Seungjin Lee, Changjo Kim, In-Ho Bae and Se-Woong Baek**

Supporting Information

Colloidal InAs quantum dot-based infrared optoelectronics enabled by universal dual-ligand passivation

*Min-Jae Si, Seungin Jee, Minjung Yang, Dongeon Kim, Yongnam Ahn, Seungjin Lee, Se-Woong Baek**

Min-Jae Si, Seungin Jee, Minjung Yang, Dongeon Kim, Yongnam Ahn, Se-Woong Baek
Department of Chemical and Biological Engineering, Korea University, Seoul, 02841 Republic of Koreagar

Seungjin Lee
Department of Energy Engineering, Korea Institute of Energy Technology (KENTECH), Naju, 58330 Republic of Korea

E-mail: sewoongbaek@korea.ac.kr

Keywords: colloidal quantum dots, photodetector, infrared, photomultiplication, surface passivation

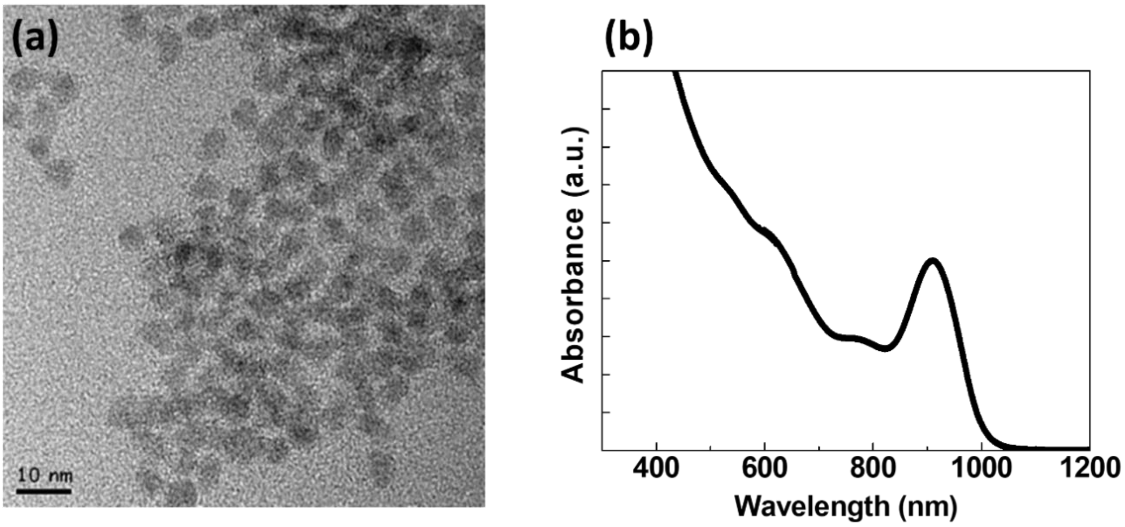

Figure S1. Pristine InAs CQDs used for optoelectronic devices. (a) TEM image of pristine InAs CQDs (b) Normalized absorption spectrum of pristine InAs CQD ink capped with OA ligands after synthesis. The first excitonic peak wavelength : 930 nm

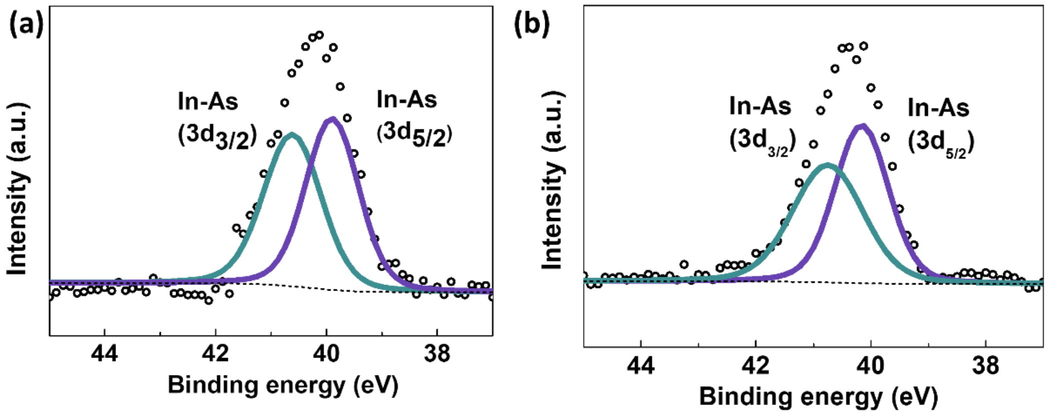

Figure S2. **Comparison of XPS As 3d spectra of InAs CQD.** (a) pristine OA ligand-capped CQD (b) ET ligands-capped CQD using direct ligand exchange (Method I). 0.3M of ET ligands in N,N-dimethylformamide(DMF) were added to the InAs CQD for direct ligand exchange. Both CQDs exhibit only In-As bonds in XPS spectra of As3d. Distinctive As-S bonds are not observed near 43 eV after direct ligand exchange with ET ligands, which indicates that sulfur atoms are barely bonded to the surface of InAs CQDs.

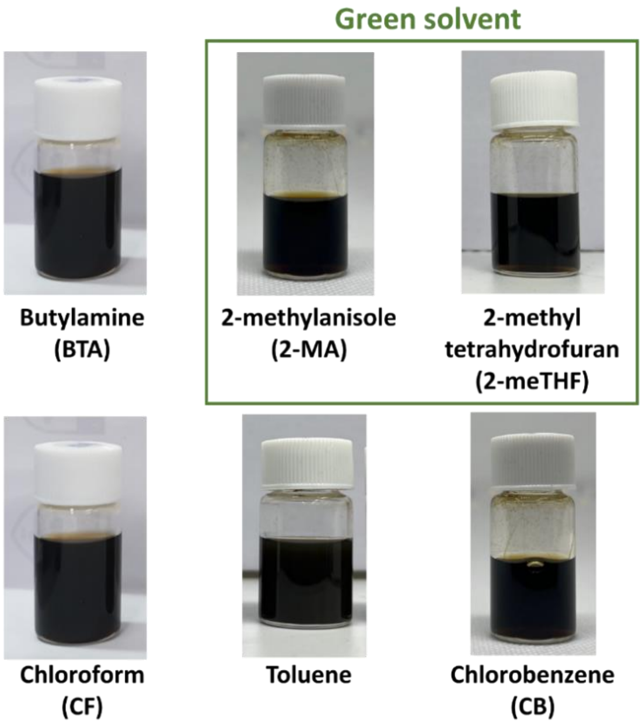

Figure S3. Photograph images displaying ET-exchanged InAs CQDs dissolved in various solvents using IPT method.

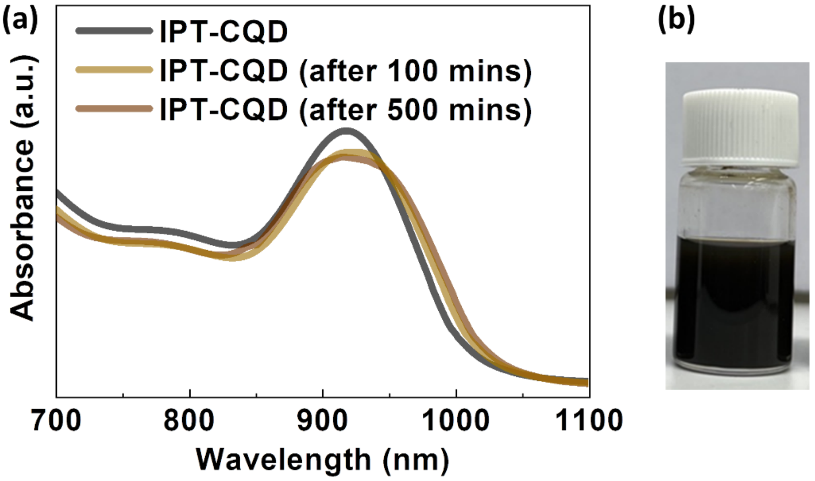

Figure S4. Colloidal stability of ET-CQD ink exchanged by IPT method. (a) Absorbance spectrum of ET ligand exchanged CQDs using IPT method (black), after 100 mins (yellow brown), and after 500 mins (brown) (b) Photographic image of CQD ink after 500 mins. CQD ink remained stable colloidal state after 500 mins and the absorption spectrum only exhibits 9.8% of intensity loss.

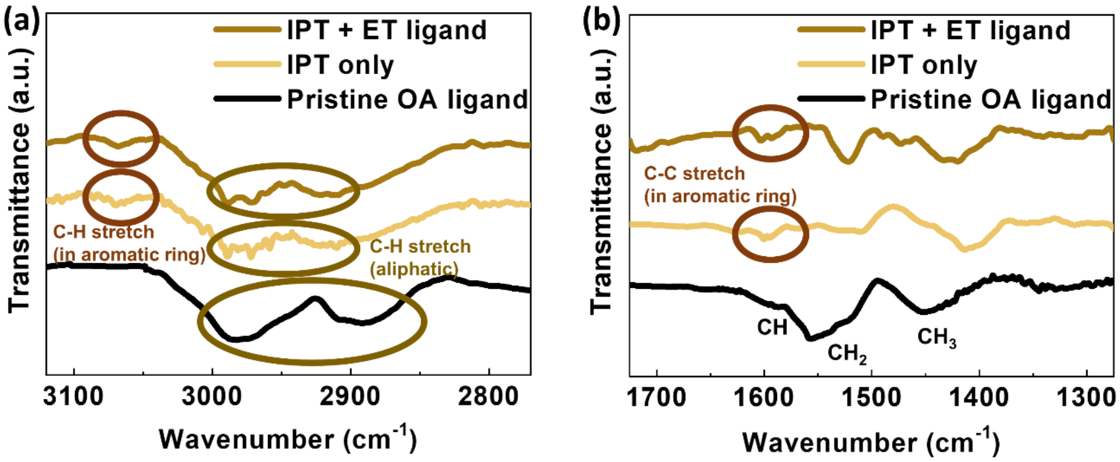

Figure S5. FTIR spectra of InAs CQD solids. (a), (b) ET ligands using IPT method (yellow brown), IPT treatment before adding ligands (yellow), and pristine OA ligands (black). InAs CQD solids using IPT method exhibit aromatic C-H and C-C stretch near 3050 cm^{-1} and 1600 cm^{-1} because of the BA ligand. The peaks indicating aliphatic C-H stretch also dramatically reduce after IPT and ET ligand exchange, which implies the removal of OA ligands..

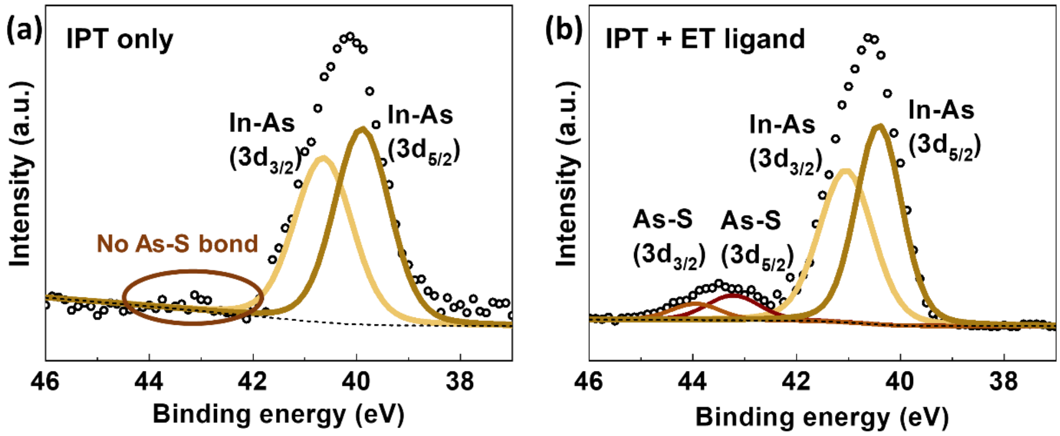

Figure S6. XPS spectra of InAs CQD solids treated with (a) only IPT and (b) ET ligands after IPT method. A peak near 43.5 eV appears which corresponds to As-S bond after 0.3 M of ET ligands were inserted dropwise. A peak showing the As-S bond indicates that the thiol ligands form a covalent bond with As atoms and passivate the surface of CQDs.

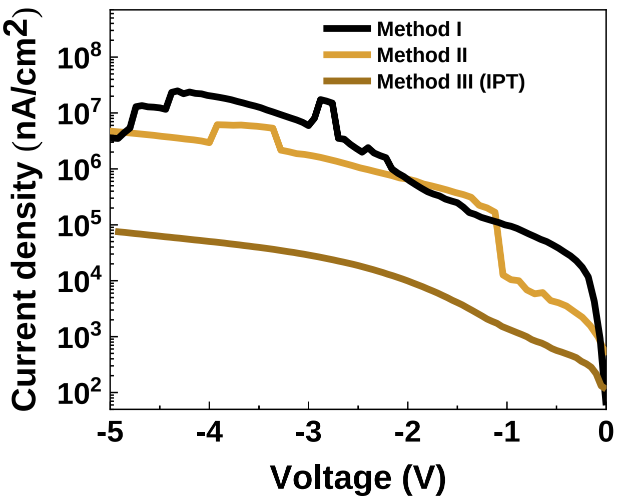

Figure S7. Dark *J*-*V* characteristics of InAs CQD photodiodes using each ligand exchange method: method I (black), method II (yellow brown), and method III (brown)

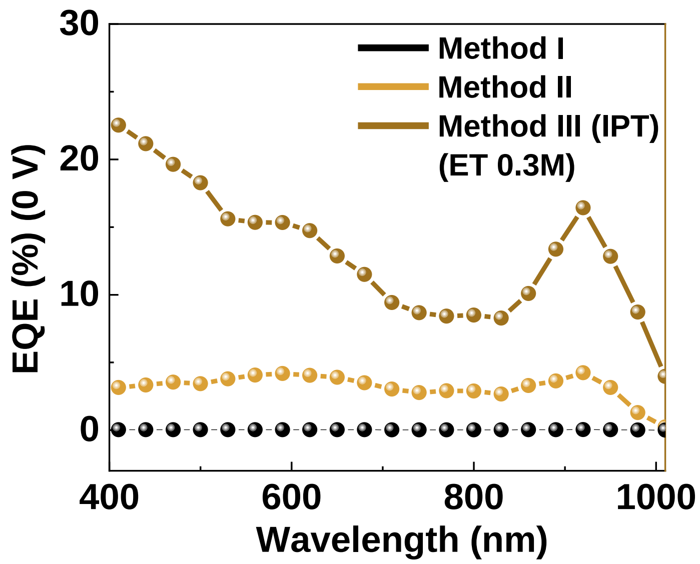

Figure S8. EQE spectra of CQD device at 0 V with method I (black), method II (yellow brown), and method III (brown). An ET ligand concentration of 0.3 M was employed for IPT process.

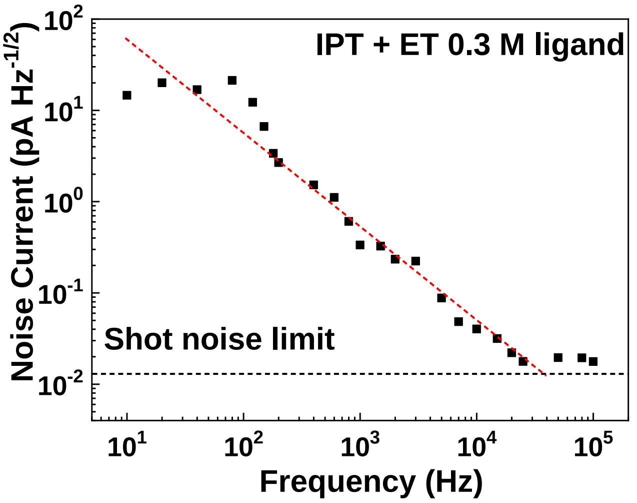

Figure S9. Noise current of photodiode based on ET-CQD using IPT method with different operating frequency. The noise current was measured using current pre-amplifier and lock-in amplifier. The noise current logarithmically decreases with increasing frequency and converges at the value of 0.018 pA $Hz^{-1/2}$, which is about 50% above the value of shot-noise limit. The frequency-dependent noise current originates from the flicker noise [1] which is typically exhibited in CQD-based optoelectronics. [2]

The shot-noise limit was measured using eq. 1

$$I_{shot} = (2qI_d)^{1/2} \text{ (eq. 1)}$$

where q is the electric charge, 1.60×10^{-19} C, and I_d stands form dark current.

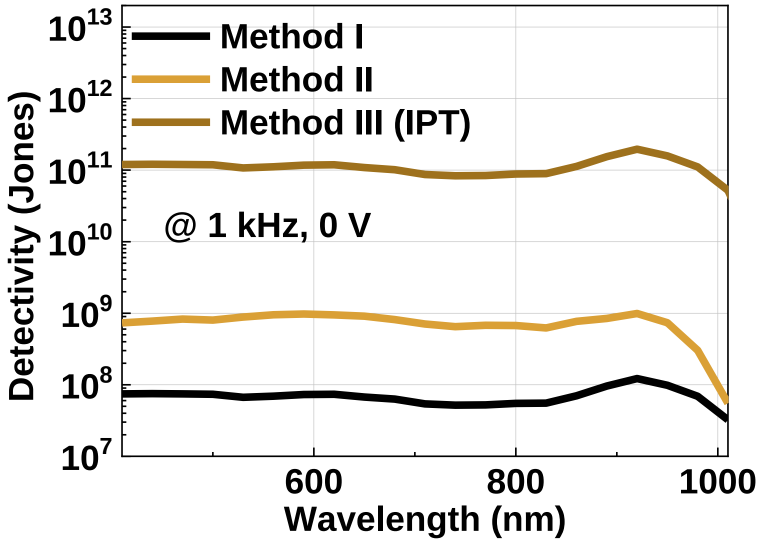

Figure S10. Specific detectivity of CQD device based on ligand exchange methods: method I (black), method II (yellow-brown), and method III (brown).

Applied voltage (V)	Responsivity (A/W)	Rise time (ns)	Fall time (ns)
0	0.066	41.3	112
-1	0.140	36.7	87.7
-2	0.408	26.8	73,1
-3	0.844	23.1	58.5
-4	1.377	15.7	49.3
-5	2.192	12.4	36.3

Table S1. Photodetector performances depending on applied voltage.

Supplementary Note 1. Determination of trap densities of the devices using space-charge-limited current (SCLC) measurement.

Bias induced trap filling enables trap-filled region over forward bias of V_{TFL}. Trap density of the device can be calculated from the voltage where the trap-filled region started using the following equation. [3]

$$N = \left(\frac{2\varepsilon\varepsilon_0}{eL^2}\right) V_{TFL} \quad \text{(eq. 2)}$$

In the formula, ε is the dielectric constant of InAs, A value of 12.3 was used for dielectric constant for InAs CQDs. [4], ε_0 is the vacuum permittivity (8.854 × 10^{-12} F/m), e is the elementary charge carried by a single electron (1.602 × 10^{-19} C), L is the thickness of CQD active layer (~120 nm), and V_{TFL} is trap-filled limit voltage. We measured the thickness of the active layer by SEM and calculated the trap density using V_{TFL}.

Table S2. V_{TFL} and calculated trap density dependent on photodiode device structure.

ET ligand concentration (M)	V_{TFL} (V)	Trap density (/cm^3)
0.1 M	0.275	2.60 × 10^{16}
0.3 M	0.243	2.29 × 10^{16}

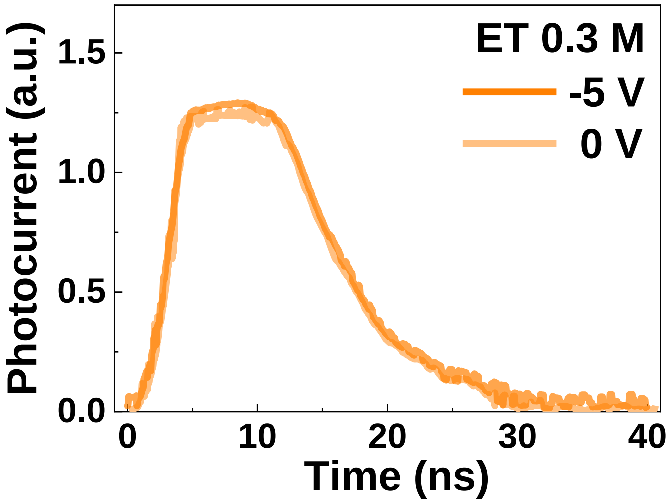

Figure S11. Transient photoresponse of IPT-treated InAs CQD photodiodes (ET ligands concentration: 0.3M) with 0V (light orange) and -5V bias (orange).

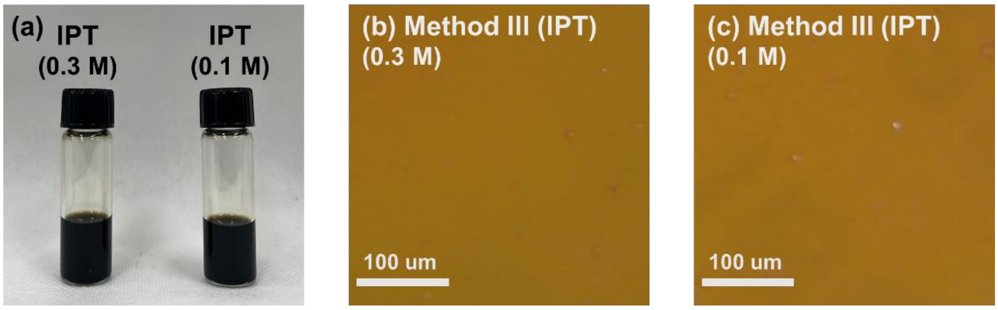

Figure S12. Photographic images of CQD ink using IPT process with ET concentrations of (a) 0.3 M (left) and 0.1 M (right). Optical microscope images of CQD thin-films fabricated using the CQD inks with ET concentrations of (b) 0.3 M and (c) 0.1 M.

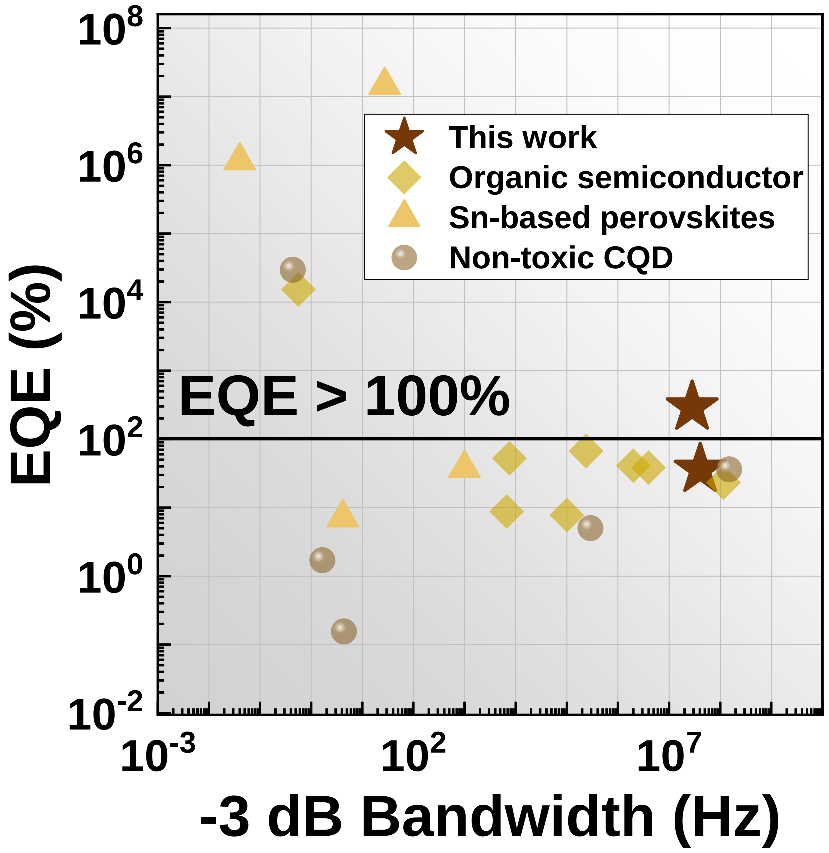

Figure S13. EQE vs. -3dB bandwidth of the solution-processed NIR photodetectors. Non-toxic CQDs (grey circle), Pb-free perovskites (orange triangle), organic semiconductors (yellow square) and this work (brown pentacle). -3dB bandwidth were calculated using rise time.

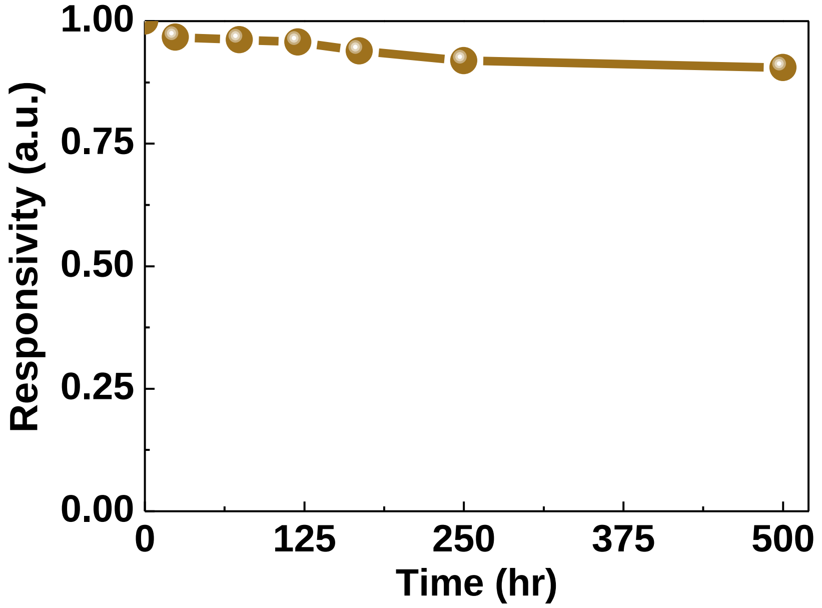

Figure S14. Photodetector stability of based on IPT-InAs CQDs measured by time-dependent responsivity. The responsivity of the photodiode was calculated with EQE spectra which was measured at -5 V in ambient air condition.

Table S3. Solution-processed NIR photodetectors using non-toxic materials.

	Device type	Photoactive material	λ (nm)	EQE (%) [bias]	Responsivity (A/W)	Rise time (ns)	Fall time (ns)	Area (mm^2)	Detectivity (Jones)	Ref.
1	PD	PTT:PCBM	850	38 [-5 V]	0.26	100	100	12	-	[5]
2	PD	PBTTT:PCBM	960	7.75 [-1 V]	0.06	1160	-	20	4.8×10^{12}	[6]
3	PD	Cyanine dye	850	23 [-2 V]	0.157	-	1000	160	3.0×10^{12}	[7]
4	PD	$ZnPc:C_{60}$	875	23 [0 V]	0.16	3	151	0.25	1.0×10^{11}	[8]
5	PD	PTB7TH:COi8DFIC: $PC_{71}BM$	900	40.8 [-0.5 V]	0.37	175	900	0.25	5.6×10^{11}	[9]
6	PT	$FASnI_3$	850	1.16×10^6 [0.5 V (V_{DS})]	7300	8.70×10^9	5.70×10^{10}	0.0048	1.9×10^{12}	[10]
7	PD	PTB7TH:Co1-4Cl	920	6.70×10^1 [-0.1 V]	0.5	1820	1820	-	1.0×10^{12}	[11]
8	PC	$CsSnI_3$	940	7.12 [-0.1 V]	0.054	8.38×10^7	2.43×10^8	-	3.8×10^5	[12]
8	PT	$FASnI_3$	850	1.46×10^7 [0.5 V (V_{DS})]	1.00×10^5	1.28×10^{11}	2.26×10^{11}	-	4.3×10^{12}	[13]
9	PT	$CuInSe_2$ CQD	1064	2.95×10^4 [1 V (V_{DS})]	253.2	8.00×10^8	1.50×10^9	-	2.4×10^{12}	[14]
10	PD	$CsSnI_3$	850	37.5 [0 V]	0.257	3.50×10^5	1.60×10^6	-	1.5×10^{11}	[15]
11	PD	Ag_2Se CQD/TiO_2	840	1.7 [-0.5 V]	0.0115	2.10×10^8	2.40×10^8	49	-	[16]
12	PD	InAs CQD	930	36 [-1 V]	0.27	-	65	0.8	1.6×10^{11}	[17]
13	PD	PNIR:PC71BM	850	8.75 [-0.5 V]	0.06	5.71×10^4	1.48×10^5	-	1.0×10^{10}	[18]
14	PD	P3HT:PTB7-Th:BEH	850	1.53×10^4 [-13 V]	223.2	3.58×10^8	3.58×10^8	3.8	8.8×10^{11}	[19]
14	PT	InSb CQD PCBM:poly-TPD	800	0.155 [-]	0.001	8.00×10^7	8.00×10^7	1.63×10^{-4}	1.5×10^6	[20]
15	PD	InAs CQD	940	36 [-1 V]	0.27	2.3	2	0.03	1.0×10^{11}	[21]
16	PD	In(As,P) CQDs	1140	5 [-4 V]	-	1200	600	13	1.1×10^{10}	[22]
17	PD	PBDB-T:FM2	880	52.4 [0 V]	0.372	4.90×10^4	1.40×10^4	8	4.6×10^{13}	[23]
18	PD	InAs CQD	950	37 [0 V]	0.283	1000	1400	9.8	1.9×10^{11}	[24]
18	PD	InAs CQD	920	292 [-5 V]	2.167	12.4	36	0.03	1.1×10^{10}	This work
				36 [-1 V]	0.267	8.9	14		1.9×10^{11}	

References

[1] F. P. García de Arquer, X. Gong, R. P. Sabatini, M. Liu, G.-H. Kim, B. R. Sutherland, O. Voznyy, J. Xu, Y. Pang, S. Hoogland, D. Sinton, E. Sargent, *Nature Communications* **2017**, 8, 14757.

[2] H. Liu, E. Lhuillier, P. Guyot-Sionnest, *Journal of Applied Physics* **2014**, 115, 154309.

[3] a)Y. Park, H. Kim, D. Shin, T. Kim, M. Choi, J. Kim, D. C. Lee, S. Jeong, *Advanced Optical Materials* **2022**, 10, 2201086; b)M. Biondi, M.-J. Choi, Z. Wang, M. Wei, S. Lee, H. Choubisa, L. K. Sagar, B. Sun, S.-W. Baek, B. Chen, P. Todorović, A. M. Najarian, A. Sedighian Rasouli, D.-H. Nam, M. Vafaie, Y. C. Li, K. Bertens, S. Hoogland, O. Voznyy, F. P. García de Arquer, E. H. Sargent, *Advanced Materials* **2021**, 33, 2101056.

[4] a)F. Mezrag, N. Bouarissa, M. Boucenna, *Optik* **2016**, 127, 1167; b)D. E. Aspnes, A. Studna, *Phys. Rev. B* **1983**, 27.

[5] Y. Yao, Y. Liang, V. Shrotriya, S. Xiao, L. Yu, Y. Yang, *Advanced Materials* **2007**, 19, 3979.

[6] A. Armin, R. D. Jansen-van Vuuren, N. Kopidakis, P. L. Burn, P. Meredith, *Nature Communications* **2015**, 6, 6343.

[7] H. Zhang, S. Jenatsch, J. De Jonghe, F. Nüesch, R. Steim, A. C. Véron, R. Hany, *Scientific Reports* **2015**, 5, 9439.

[8] B. Siegmund, A. Mischok, J. Benduhn, O. Zeika, S. Ullbrich, F. Nehm, M. Böhm, D. Spoltore, H. Fröb, C. Körner, K. Leo, K. Vandewal, *Nature Communications* **2017**, 8, 15421.

[9] W. Li, Y. Xu, X. Meng, Z. Xiao, R. Li, L. Jiang, L. Cui, M. Zheng, C. Liu, L. Ding, Q. Lin, *Advanced Functional Materials* **2019**, 29, 1808948.

[10] C.-K. Liu, Q. Tai, N. Wang, G. Tang, H.-L. Loi, F. Yan, *Advanced Science* **2019**, 6, 1900751.

[11] J. Huang, J. Lee, J. Vollbrecht, V. V. Brus, A. L. Dixon, D. X. Cao, Z. Zhu, Z. Du, H. Wang, K. Cho, G. C. Bazan, T.-Q. Nguyen, *Advanced Materials* **2020**, 32, 1906027.

[12] M. Han, J. Sun, M. Peng, N. Han, Z. Chen, D. Liu, Y. Guo, S. Zhao, C. Shan, T. Xu, X. Hao, W. Hu, Z.-x. Yang, *The Journal of Physical Chemistry C* **2019**, 123, 17566.

[13] C.-K. Liu, Q. Tai, N. Wang, G. Tang, Z. Hu, F. Yan, *ACS Applied Materials & Interfaces* **2020**, 12, 18769.

[14] T. Shen, F. Li, Z. Zhang, L. Xu, J. Qi, *ACS Applied Materials & Interfaces* **2020**, 12, 54927.

[15] F. Cao, W. Tian, M. Wang, M. Wang, L. Li, *InfoMat* **2020**, 2, 577.

[16] N. Graddage, J. Ouyang, J. Lu, T.-Y. Chu, Y. Zhang, Z. Li, X. Wu, P. R. L. Malenfant, Y. Tao, *ACS Applied Nano Materials* **2020**, 3, 12209.

[17] M.-J. Choi, L. K. Sagar, B. Sun, M. Biondi, S. Lee, A. M. Najjariyan, L. Levina, F. P. García de Arquer, E. H. Sargent, *Nano Letters* **2021**, 21, 6057.

[18] H. J. Eun, H. Kye, D. Kim, I. S. Jin, J. W. Jung, S.-J. Ko, J. Heo, B.-G. Kim, J. H. Kim, *ACS Applied Materials & Interfaces* **2021**, 13, 11144.

[19] M. Liu, J. Wang, Z. Zhao, K. Yang, P. Durand, F. Ceugniet, G. Ulrich, L. Niu, Y. Ma, N. Leclerc, X. Ma, L. Shen, F. Zhang, *The Journal of Physical Chemistry Letters* **2021**, 12, 2937.

[20] M. He, Z. Xu, S.-W. Zhang, M. Zhang, C. Wu, B. Li, J. Li, L. Wang, S. Zhao, F. Kang, G. Wei, *Advanced Photonics Research* **2022**, 3, 2100305.

[21] B. Sun, A. M. Najarian, L. K. Sagar, M. Biondi, M.-J. Choi, X. Li, L. Levina, S.-W. Baek, C. Zheng, S. Lee, A. R. Kirmani, R. Sabatini, J. Abed, M. Liu, M. Vafaie, P. Li, L. J. Richter, O. Voznyy, M. Chekini, Z.-H. Lu, F. P. García de Arquer, E. H. Sargent, *Advanced Materials* **2022**, 34, 2203039.

[22] J. Leemans, V. Pejović, E. Georgitzikis, M. Minjauw, A. B. Siddik, Y.-H. Deng, Y. Kuang, G. Roelkens, C. Detavernier, I. Lieberman, P. E. Malinowski, D. Cheyns, Z. Hens, *Advanced Science* **2022**, 9, 2200844.

[23] Y. Zhang, Y. Yu, X. Liu, J. Miao, Y. Han, J. Liu, L. Wang, *Advanced Materials* **2023**, n/a, 2211714.

[24] P. Xia, B. Sun, M. Biondi, J. Xu, O. Atan, M. Imran, Y. Hassan, Y. Liu, J. M. Pina, A. M. Najarian, L. Grater, K. Bertens, L. K. Sagar, H. Anwar, M.-J. Choi, Y. Zhang, M. Hasham, F. P. G. de Arquer, S. Hoogland, M. W. B. Wilson, E. H. Sargent, *Advanced Materials*, n/a, 2301842.
